# Several epidemic and multiple sporadic genotypes of OXA-244-producing *Escherichia coli* in Poland; predominance of the ST38 clone

**DOI:** 10.1007/s10096-024-04954-0

**Published:** 2024-10-07

**Authors:** Marta Biedrzycka, Radosław Izdebski, Marek Gniadkowski, Dorota Żabicka

**Affiliations:** 1 Department of Molecular Microbiology, National Medicines Institute, Chełmska 30/34, Warsaw, 00-725 Poland; 2 Department of Epidemiology and Clinical Microbiology, National Reference Centre for Susceptibility Testing, National Medicines Institute, Chełmska 30/34, Warsaw, 00-725 Poland

**Keywords:** OXA-244, *Escherichia coli*, ST38, Poland

## Abstract

OXA-244-producing *Escherichia coli* (OXA-244-Ec) has disseminated in Europe, mostly in the community. In Poland it has spread since 2017, especially in 2023, but in contrast to other countries, all isolates have been identified in hospitals so far. The isolates (*n* = 101) represented one large and two limited outbreaks in different regions, and multiple epidemiologically and genetically non-related organisms. The OXA-244-Ec population consisted of 14 STs, with ST38 dominating. The ST38 isolates belonged to two major lineages, Clusters A and B, responsible for two of the hospital outbreaks. Enhanced concern and vigilance are necessary in the OXA-244-Ec surveillance.

**Supplementary Information:**

The online version contains supplementary material available at 10.1007/s10096-024-04954-0.

*Escherichia coli* with oxacillinase (OXA)-244 (OXA-244-Ec) has become an important type of carbapenemase-producing Enterobacterales (CPE). Reported from 2013, since 2017/18 it has spread across Europe, mainly in the community, creating a potential risk of large-scale carbapenem resistance dissemination [1]. In part this ‘success’ is due to detection difficulties, arising from weak carbapenemase activity of OXA-244 (a point-mutant derivative of OXA-48), and low-level resistance of the enzyme producers [1]. Most of the OXA-244-Ec isolates belong to sequence type (ST) 38, forming then two lineages with specific molecular markers, defined by ECDC as Clusters A and B [1]. Cluster A, more recent and homogeneous, has spread since 2016 in the community in Germany [2], France [3], Switzerland [4] or The Netherlands [5], and in 2020 caused a hospital outbreak in Norway [6]. Otherwise, in 2023 Poland recorded a nosocomial outbreak of Cluster B, observed from 2013 and being more diversified [7].

## Screening for OXA-244-Ec

In February 2023, the OXA-244-Ec ST38 outbreak commenced in hospital HF1 in western Poland, region Lubuskie [7]. Problems with its control prompted the National Reference Centre for Susceptibility Testing (NRCST), responsible for all-country CPE monitoring, to perform a specific OXA-244-Ec survey. All OXA-48-type-producing *E. coli* (OXA-48-type-Ec) isolates ever confirmed by the NRCST by the end of 2023 were screened for the *bla*_OXA−244_ gene by FspBI digestion of *bla*_OXA−48_-like amplicons, as described previously [7]. Subsequently, all OXA-244-Ec isolates were sequenced with MiSeq (Illumina, San Diego, USA) as reported [7]. Thirty-eight OXA-244-Ec ST38 isolates of the HF1 outbreak, sequenced and analysed previously [7], were included for the complete view of OXA-244-Ec epidemiology in Poland.

## Epidemiology, clonality and phylogeny of OXA-244-Ec

**Fig. 1:**
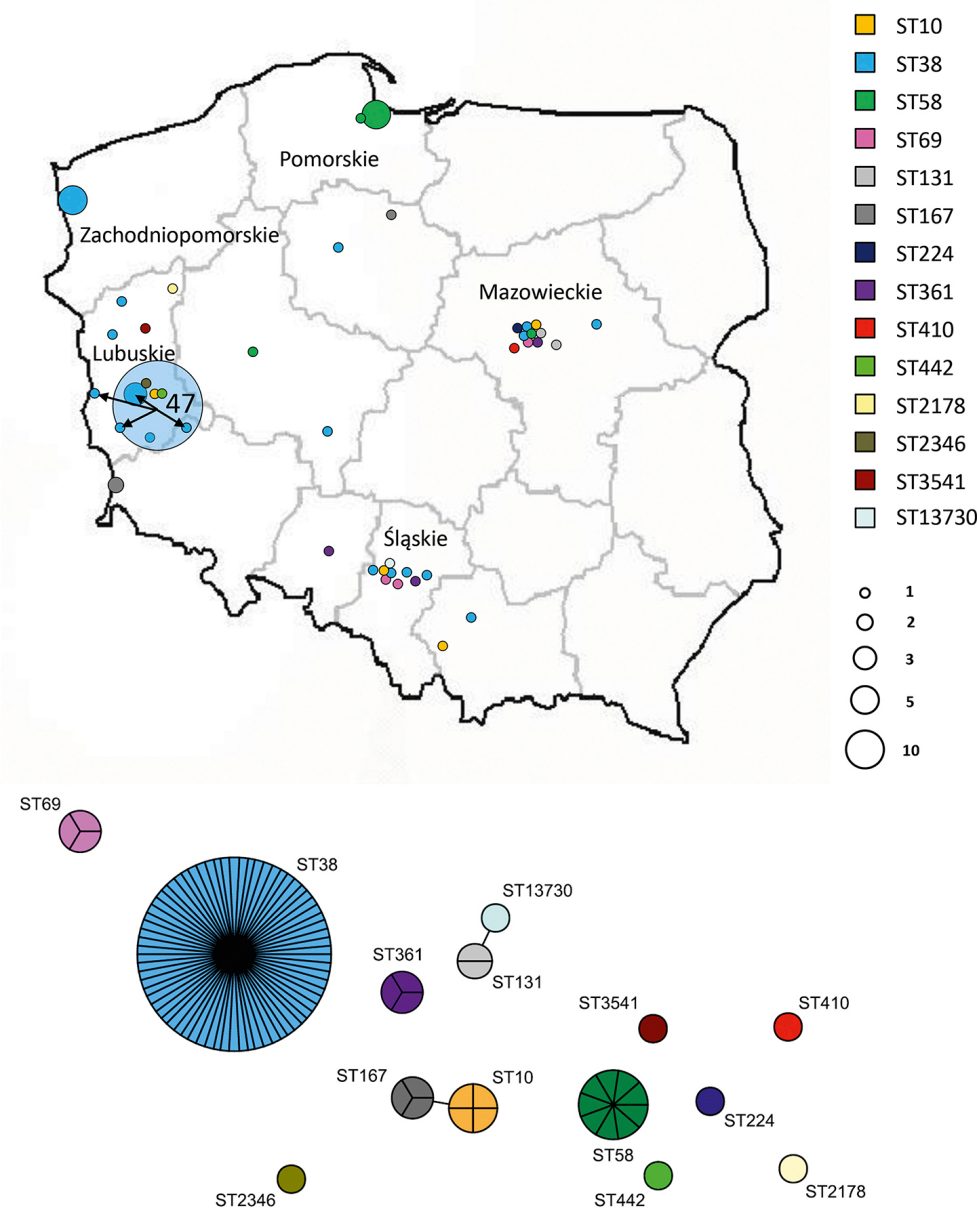
Geographic distribution and clonal analysis of OXA-244-Ec isolates in Poland. Geographic distribution of the isolates is shown in the upper part; administrative regions with multiple cases are labelled. Circles represent medical centres where the isolates were recorded. Sizes of the circles are proportional to numbers of cases, and colours refer to individual STs. The dot corresponding to hospital HF1 is transparent in order not to mask other centres in the Lubuskie region. OXA-244-Ec population structure is shown in the bottom part of the figure. The minimum-spanning tree was constructed on the basis of 7-loci MLST data. Each circle represents one ST, and each fragment of a pie chart corresponds to one isolate. The size of a circle is proportional to the number of isolates of this ST. Connecting lines infer phylogenetic relatedness in terms of allelic differences; thick solid line indicates a single-locus variant

Altogether, 101 OXA-244-Ec isolates from December 2017 to December 2023 were analysed, including 20 isolates from 2017 to 22 and 81 isolates from 2023 (Table 1). These constituted 41.4% of all OXA-48-type-Ec recorded in Poland since 2013 (*n* = 244) (Figure S1). The OXA-244-Ec isolates were identified in 38 hospitals in 29 cities of 10/16 administrative regions, five of which recorded multiple isolates, including Lubuskie, Pomorskie and Zachodniopomorskie (Fig. 1). Isolates were collected from male (62%) and female (38%) patients, with the mean age of 63.5 years (5 days – 97 years). Most of the isolates (88%) were from colonization (rectal mostly).

**Table 1:** Epidemiologic data and SNP scores between OXA-244-producing *Escherichia coli* isolates

ST	Isolate	Centre	Region	Isolation date	Sex	Age	Isolation source	SNPs	Comment
ST38	5550/23^*a*^	HF1	Lubuskie	2023-02-27	M	71 years	rectal swab	0	ST38 cluster B; regional outbreak
	5551/23	HF1	Lubuskie	2023-02-27	M	74 years	rectal swab	0	ST38 cluster B; regional outbreak
	5553/23	HF1	Lubuskie	2023-03-03	M	69 years	rectal swab	0	ST38 cluster B; regional outbreak
	5554/23	HF1	Lubuskie	2023-03-03	M	75 years	rectal swab	0	ST38 cluster B; regional outbreak
	2080/23	HF1	Lubuskie	2023-03-03	M	69 years	rectal swab	0	ST38 cluster B; regional outbreak
	5555/23	HF1	Lubuskie	2023-03-06	M	97 years	rectal swab	0	ST38 cluster B; regional outbreak
	5556/23	HF1	Lubuskie	2023-03-08	M	72 years	rectal swab	0	ST38 cluster B; regional outbreak
	5557/23	HF1	Lubuskie	2023-03-08	M	43 years	rectal swab	0	ST38 cluster B; regional outbreak
	5561/23	HF1	Lubuskie	2023-05-15	F	72 years	rectal swab	0	ST38 cluster B; regional outbreak
	5562/23	HF1	Lubuskie	2023-05-15	F	82 years	rectal swab	0	ST38 cluster B; regional outbreak
	5564/23	HF1	Lubuskie	2023-05-17	F	33 years	rectal swab	0	ST38 cluster B; regional outbreak
	5565/23	HF1	Lubuskie	2023-05-17	M	70 years	rectal swab	0	ST38 cluster B; regional outbreak
	5566/23	HF1	Lubuskie	2023-05-17	F	65 years	rectal swab	0	ST38 cluster B; regional outbreak
	5568/23	HF1	Lubuskie	2023-05-19	M	82 years	rectal swab	0	ST38 cluster B; regional outbreak
	5572/23	HF1	Lubuskie	2023-05-30	M	55 years	rectal swab	0	ST38 cluster B; regional outbreak
	5573/23	HF1	Lubuskie	2023-06-01	F	2 years	rectal swab	0	ST38 cluster B; regional outbreak
	5575/23	HF1	Lubuskie	2023-06-06	M	67 years	rectal swab	0	ST38 cluster B; regional outbreak
	5578/23	HF1	Lubuskie	2023-06-09	M	59 years	rectal swab	0	ST38 cluster B; regional outbreak
	5582/23	HF1	Lubuskie	2023-06-16	M	74 years	rectal swab	0	ST38 cluster B; regional outbreak
	5585/23	HF1	Lubuskie	2023-06-21	M	52 years	rectal swab	0	ST38 cluster B; regional outbreak
	6227/23	HF2	Lubuskie	2023-07-11	F	-	rectal swab	0	ST38 cluster B; regional outbreak
	6228/23	HF2	Lubuskie	2023-07-03	M	-	rectal swab	0	ST38 cluster B; regional outbreak
	7535/23	HF1	Lubuskie	2023-09-03	F	30 years	rectal swab	0	ST38 cluster B; regional outbreak
	8601/23	HF8	Lubuskie	2023-10-11	M	82 years	rectal swab	0	ST38 cluster B; regional outbreak
	5552/23	HF1	Lubuskie	2023-02-28	F	76 years	rectal swab	1	ST38 cluster B; regional outbreak
	5559/23	HF1	Lubuskie	2023-03-22	F	82 years	rectal swab	1	ST38 cluster B; regional outbreak
	5563/23	HF1	Lubuskie	2023-05-15	M	83 years	rectal swab	1	ST38 cluster B; regional outbreak
	5567/23	HF1	Lubuskie	2023-05-18	M	56 years	rectal swab	1	ST38 cluster B; regional outbreak
	5569/23	HF1	Lubuskie	2023-05-22	M	70 years	rectal swab	1	ST38 cluster B; regional outbreak
	5570/23	HF1	Lubuskie	2023-05-29	F	64 years	rectal swab	1	ST38 cluster B; regional outbreak
	5574/23	HF1	Lubuskie	2023-06-05	M	70 years	rectal swab	1	ST38 cluster B; regional outbreak
	5576/23	HF1	Lubuskie	2023-06-06	M	83 years	rectal swab	1	ST38 cluster B; regional outbreak
	5580/23	HF1	Lubuskie	2023-06-13	M	66 years	rectal swab	1	ST38 cluster B; regional outbreak
	5581/23	HF1	Lubuskie	2023-06-15	F	72 years	rectal swab	1	ST38 cluster B; regional outbreak
	5615/23	HF5	Lubuskie	2023-06-29	F	58 years	rectal swab	1	ST38 cluster B; regional outbreak
	6961/23	HF1	Lubuskie	2023-08-08	M	71 years	rectal swab	1	ST38 cluster B; regional outbreak
	8493/23	HF1	Lubuskie	2023-10-13	M	69 years	rectal swab	1	ST38 cluster B; regional outbreak
	2082/23	HF1	Lubuskie	2023-03-03	M	65 years	rectal swab	2	ST38 cluster B; regional outbreak
	5558/23	HF1	Lubuskie	2023-03-10	M	26 years	rectal swab	2	ST38 cluster B; regional outbreak
	5571/23	HF1	Lubuskie	2023-05-29	F	85 years	rectal swab	2	ST38 cluster B; regional outbreak
	5577/23	HF1	Lubuskie	2023-06-06	M	80 years	rectal swab	2	ST38 cluster B; regional outbreak
	6094/23	HF1	Lubuskie	2023-07-13	F	78 years	rectal swab	2	ST38 cluster B; regional outbreak
	6150/23	HD2	Dolnośląskie	2023-07-11	M	43 years	rectal swab	2	ST38 cluster B; regional outbreak
	5579/23	HF1	Lubuskie	2023-06-10	M	70 years	rectal swab	3	ST38 cluster B; regional outbreak
	6378/23	HF6	Lubuskie	2023-07-29	M	70 years	rectal swab	3	ST38 cluster B; regional outbreak
	7536/23	HF1	Lubuskie	2023-08-29	M	67 years	rectal swab	3	ST38 cluster B; regional outbreak
	7539/23	HF1	Lubuskie	2023-08-31	F	92 years	rectal swab	3	ST38 cluster B; regional outbreak
	8495/23	HF1	Lubuskie	2023-09-27	F	55 years	rectal swab	3	ST38 cluster B; regional outbreak
	5584/23	HF1	Lubuskie	2023-06-20	M	72 years	rectal swab	4	ST38 cluster B; regional outbreak
	8496/23	HF1	Lubuskie	2023-09-22	M	79 years	rectal swab	4	ST38 cluster B; regional outbreak
	9247/23	HF9	Lubuskie	2023-11-03	F	68 years	rectal swab	4	ST38 cluster B; regional outbreak
	5583/23	HF1	Lubuskie	2023-06-19	M	83 years	rectal swab	5	ST38 cluster B; regional outbreak
	5560/23	HF1	Lubuskie	2023-04-07	F	69 years	rectal swab	9	ST38 cluster B; regional outbreak
	2213/18	HW1	Mazowieckie	2018-03-24	M	65 years	rectal swab	1073	ST38 cluster B; single case
	8517/18	HP1	Wielkopolskie	2018-11-22	M	72 years	rectal swab	1134	ST38 cluster B; single case
	2050/21	HC1	Kujawsko-Pomorskie	2021-03-23	M	73 years	wound	1143	ST38 cluster B; single case
	9336/23	HS7	Śląskie	2023-10-30	F	74 years	wound	1792	ST38 cluster B; single case
	7712/23	HS5	Śląskie	2023-09-04	F	66 years	urine	2979	ST38 cluster B; single case
	1505/20	HZ1	Zachodniopomorskie	2020-03-02	F	9 days	rectal swab	3309	ST38 cluster A; hospital outbreak
	1506/20	HZ1	Zachodniopomorskie	2020-02-28	M	5 days	rectal swab	3308	ST38 cluster A; hospital outbreak
	1507/20	HZ1	Zachodniopomorskie	2020-03-03	M	8 days	rectal swab	3312	ST38 cluster A; hospital outbreak
	1504/20	HZ1	Zachodniopomorskie	2020-02-29	F	23 years	rectal swab	3314	ST38 cluster A; hospital outbreak
	8934/17	HK1	Małopolskie	2017-12-09	F	49 years	urine	3287	ST38 cluster A; single case
	6954/19	HW2	Mazowieckie	2019-08-24	M	41 years	urine	3292	ST38 cluster A; single case
	6607/22	HS1	Śląskie	2022-07-21	M	73 years	rectal swab	3315	ST38 cluster A; single case
	6000/23	HS2	Śląskie	2023-07-06	M	-	wound	3317	ST38 cluster A; single case
	10,134/19	HF2	Lubuskie	2019-12-15	M	80 years	rectal swab	3318	ST38 cluster A; single case
	6093/23	HF1	Lubuskie	2023-06-29	F	76 years	rectal swab	3330	ST38 cluster A; single case
	4587/23	HW8	Mazowieckie	2023-05-30	F	5 years	rectal swab	3323	ST38 cluster A; single case
	810/23	HF7	Lubuskie	2023-01-17	M	75 years	rectal swab	3334	ST38 cluster A; single case
ST58	6864/23 ^*a*^	HG1	Pomorskie	2023-07-19	F	56 years	pharyngeal swab	0	city outbreak
	6863/23	HG1	Pomorskie	2023-07-25	M	85 years	urine	1	city outbreak
	6865/23	HG1	Pomorskie	2023-08-02	M	-	blood	1	city outbreak
	6866/23	HG1	Pomorskie	2023-07-27	M	66 years	rectal swab	2	city outbreak
	6868/23	HG1	Pomorskie	2023-07-25	F	70 years	rectal swab	2	city outbreak
	6869/23	HG2	Pomorskie	2023-07-20	F	63 years	rectal swab	2	city outbreak
	6867/23	HG1	Pomorskie	2023-07-28	F	69 years	rectal swab	3	city outbreak
	6283/23	HW9	Mazowieckie	2023-07-05	M	92 years	rectal swab	199	single case
	10,338/23	HP2	Wielkopolskie	2023-12-15	M	77 years	rectal swab	206	single case
ST10	1527/23 ^*a*^	HK2	Małopolskie	2023-02-07	M	77 years	rectal swab	0	single case
	8886/23	HW11	Mazowieckie	2023-10-24	M	47 years	wound	208	single case
	8494/23	HF1	Lubuskie	2023-09-13	F	60 years	rectal swab	5007	single case
	6988/23	HS3	Śląskie	2023-08-11	M	79 years	rectal swab	6618	single case
ST69	9537/23 ^*a*^	HS3	Śląskie	2023-11-06	M	76 years	rectal swab	0	single case
	9985/23	HW3	Mazowieckie	2019-12-09	F	89 years	rectal swab	4043	single case
	9465/23	HS8	Śląskie	2023-11-08	M	61 years	rectal swab	4091	single case
ST167	1067/22 ^*a*^	HC2	Kujawsko-Pomorskie	2022-01-21	-	62 years	rectal swab	0	single case
	8254/22	HD1	Dolnośląskie	2022-09-15	F	84 years	BAL	5234	hospital dissemination
	8724/22	HD1	Dolnośląskie	2022-10-07	F	73 years	rectal swab	5374	hospital dissemination
ST361	7476/21 ^*a*^	HW5	Mazowieckie	2021-09-23	F	35 years	cervical swab	0	single case
	9687/23	HS4	Śląskie	2023-08-10	M	71 years	rectal swab	576	local dissemination
	8342/23	HS6	Śląskie	2023-09-19	F	51 years	rectal swab	587	local dissemination
ST131	5887/21 ^*a*^	HW4	Mazowieckie	2021-07-26	M	22 years	rectal swab	0	single case
	3895/23	HW7	Mazowieckie	2023-05-06	M	69 years	BAL	63	single case
ST224	10,056/21	HW6	Mazowieckie	2021-12-22	M	69 years	rectal swab	-	single case
ST410	7920/23	HW10	Mazowieckie	2023-09-16	F	90 years	urine	-	single case
ST442	1747/21	HF1	Lubuskie	2021-03-10	M	81 years	rectal swab	-	single case
ST2178	8174/23	HF3	Lubuskie	2023-09-29	M	69 years	rectal swab	-	single case
ST2346	6226/23	HF2	Lubuskie	2023-07-09	F	-	rectal swab	-	single case
ST3541	1047/21	HF4	Lubuskie	2021-02-12	F	72 years	rectal swab	-	single case
ST13730	8386/23	HS3	Śląskie	2023-10-02	M	54 years	rectal swab	-	single case

^*a*^ – reference genomes in SNP analyses

Fourteen STs were discerned using the mlst tool (https://github.com/tseemann/mlst) (Table 1; Fig. 1). ST38 dominated (*n* = 70; 69.3%), and was followed by ST58 (*n* = 9; 8.9%), ST10 (*n* = 4; 4.0%), ST69, ST167 and ST361 (*n* = 3; 3% each), and ST131 (*n* = 2; 2%). The remaining STs had single isolates. ST38 comprised the first organism identified retrospectively, being a sporadic isolate from Cracow, December 2017. By the end of 2023, OXA-244-Ec ST38 was recorded in 19 hospitals in 18 towns of eight regions (Table 1; Fig. 1). Lubuskie with the outbreak hospital HF1 [7] was the most affected area (*n* = 55; 78.6% of ST38). The clonal diversity of OXA-244-Ec has been observed also in other countries [2, 3, 5].

**Fig. 2:**
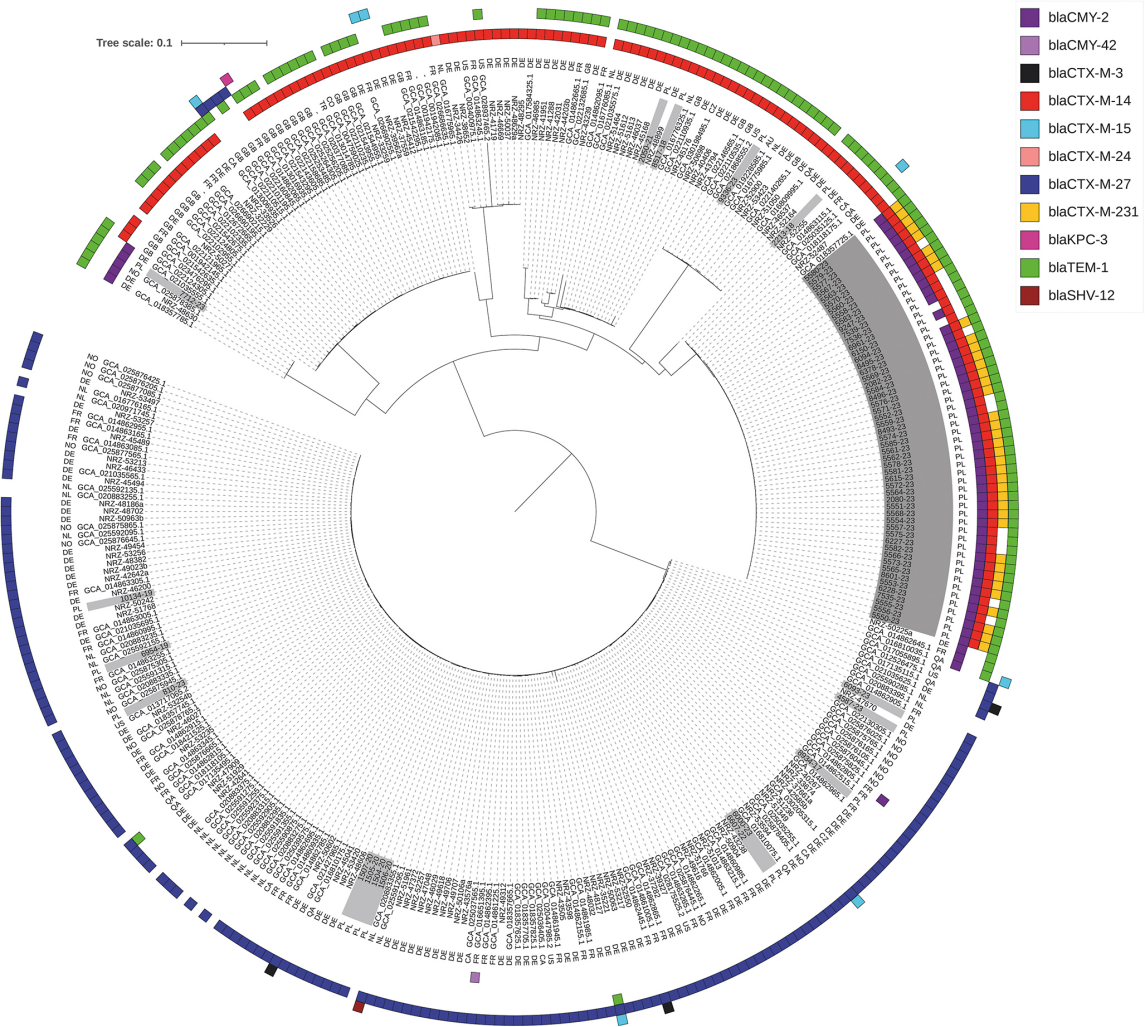
Single-nucleotide polymorphism (SNP)-based phylogenetic tree of OXA-244-Ec ST38 isolates from Poland (*n* = 70), GenBank (*n* = 164; all international isolates as of August 27, 2023) and Germany (*n* = 100) [2]. Numbers on the inner circle are the original numbers of the study isolates, GenBank assembly numbers and original numbers from the German study. Country symbols: AU, Australia; CA, Canada; CO, Colombia; CZ, Czechia; DE, Germany; FR, France; GB, United Kingdom; NL, the Netherlands; NO, Norway; PL, Poland; QA, Qatar; TR, Turkey; US, USA. The outbreak isolates are marked with dark gray, whereas the remaining Polish isolates with light gray. Coloured squares represent selected AMR genes. The tree was constructed using Parsnp and visualised with iTOL

The 70 ST38 isolates were subjected to SNP-based comparative analyses, using BioNumerics v.7.6.3 (Applied Maths NV, Sint-Martens-Latem, Belgium) with a strict SNP filtering template. The in-sample clonality analysis utilised the first HF1 outbreak isolate (isolate 5550/23) [7] as a reference. The phylogenetic analysis was done against all 164 OXA-244-Ec ST38 genomes available in GenBank (as of August 27, 2023; selected from 217,882 *E. coli* genomes) and 100 OXA-244-Ec ST38 genomes described in Germany [2]. The in-sample analysis revealed 6890 polymorphic positions within 4.3 Mb (78%) of the reference genome, and SNP numbers between any isolate and the reference was 0-3334 SNPs (Table 1). Despite this overall diversity, the majority of the isolates (*n* = 53; 75.7% of ST38; all from 2023) formed a tight cluster with 0–9 SNPs between members, representing the on-going outbreak in Lubuskie [7]. The epicentre HF1 recorded eight further isolates in July-October 2023 (*n* = 47; 67.1% in total) and exported the organism in June-November 2023 (*n* = 7) to several hospitals in the same and the neighbouring region (Table 1; Fig. 1). Four outbreak cases in these new hospitals had been treated previously (1–6 months ago) in HF1. As it was reported, the epidemic organism was classified into the European OXA-244-Ec ST38 Cluster B, marked by the extended-spectrum β-lactamase (ESBL) gene *bla*_CTX−M−14_ (Fig. 2) [1, 7]. Five other Cluster B isolates, recovered in 2018-23 in several regions, had no links to the outbreak, being distant by 1073–2979 SNPs from the reference. In the phylogenetic analysis all Cluster B isolates, including the Lubuskie outbreak, were split into five branches of the highly varied lineage, shared mostly with isolates from Germany, The Netherlands and the UK (Fig. 2).

The second ST38 fraction of isolates (*n* = 12; 17.1%) occurred in nine towns of five regions in 2017-23 (Fig. 1). Classified into the Cluster A [1], these had the marker *bla*_CTX−M−27_ ESBL gene and were notably homogeneous, differing only by 0–44 SNPs from each other (Table 1; Fig. 2). A set of four isolates with 0–1 SNPs was discerned, representing an outbreak in hospital HZ1, region Zachodniopomorskie (Table 1; Fig. 1). These were cultured in five days from three newborns (5–9 days) and one female patient (23 years) in one obstetrics and gynaecology ward. The Cluster A isolates were scattered across the large and homogeneous branch of the OXA-244-Ec ST38 phylotree, and their closest relatives were from Germany, The Netherlands, Norway and France. (Fig. 2).

Another important clone was ST58, with nine isolates from 2023, collected in four centres of three cities and regions, mostly Gdańsk in Pomorskie. Seven isolates from hospitals HG1 and HG2 showed 0–2 SNPs in between, indicating outbreak in HG1 with transmission to HG2. OXA-244-Ec ST58 has been rare, with only single, unrelated isolate from 2015 from Egypt (GenBank acc. No. GCA_024389345.1).

## Resistomes and susceptibility

The study isolates had 30 profiles of acquired antimicrobial resistance (AMR) genes (1–18 genes per isolate; Table S1), detected using AMRFinderPlus v.3.12.8 [8]. In general, broad resistomes characterised the ST38 isolates, e.g. the Lubuskie outbreak; otherwise, ST10 isolates had only 0–2 genes apart from *bla*_OXA−244_. A remarkable resistome homogeneity of isolates within outbreaks was observed, with single-gene variations present only among the isolates from Lubuskie. Except *bla*_OXA−244_, most of the isolates had genes coding for ESBLs (*bla*_CTX−M−14_, _−15_, _−27_, _−231_) and/or AmpC-like cephalosporinases (*bla*_CMY−2_, _−145_, *bla*_DHA−1_); one ST361 isolate carried a carbapenemase gene *bla*_NDM−5_. Numerous isolates had various genes of aminoglycoside-modifying enzymes and/or *qnr*-like quinolone resistance genes, mainly of limited clinical relevance. Many OXA-244-Ec isolates contained *sul* and *dfr* genes, conferring resistance to co-trimoxazole.

Chromosomal location of *bla*_OXA−244_ genes was confirmed for three isolates representing the ST38 regional (isolate 5550/23), and ST38 and ST58 hospital outbreaks (1506/20 & 6864/23, respectively), using MinION long-read sequencing (Oxford Nanopore Technologies, Oxford, UK), and Unicycler v0.4.8 hybrid assembler [9]. Genetic context of their *bla*_OXA−244_ genes consisted of a truncated Tn1999.2 transposon derivative (IS*1R*-∆IS*1999*-*bla*_OXA−244_-∆*lysR*-IS*1R*), observed previously in OXA-244-Ec ST38 in France (variant ‘G’) [3]. All the three isolates had IncFII-like plasmids, which in case of 1506/20 and 6864/23 possessed all acquired AMR genes except *bla*_OXA−244_ (Table S2).

Antimicrobial susceptibility was tested for 30 isolates representing all resistome variants (Table S1), using broth microdilution Sensititre EUGNF, GNX3F and MDRXXF plates (Thermo Fisher Scientific, Waltham, USA), the ComASP Cefiderocol test (Liofilchem, Roseto degli Abruzzi, Italy), and agar dilution for fosfomycin. Results were interpreted according to EUCAST breakpoints v.14.0. (http://eucast.org). Susceptibility of the isolates correlated well with their resistomes (Tables S1 and S3). All isolates were resistant to penicillins, and all but four were resistant to oxyimino-β-lactams (cefotaxime at least) due to ESBL and/or AmpC production. Two isolates were resistant to cefiderocol (MIC, 4 mg/L), which together with four others (MIC, 2 mg/L) had the YRIN duplication in the PBP3 protein [10, 11]. Twenty four isolates were classified as ertapenem-resistant but most of them were susceptible to imipenem and meropenem. Two isolates had increased MICs of imipenem-relebactam and meropenem-vaborbactam, and one of these, also to ceftazidime-avibactam. The isolate resistant to all these combinations was the ST361 OXA-244 + NDM-5 producer. The other one was the first OXA-244-Ec ST38 isolate from 2017, and the reason for its increased MICs of carbapenems and carbapenem-inhibitor combinations is unclear. Of non-β-lactams, the isolates were uniformly susceptible to amikacin, tigecycline, eravacycline, colistin, nitrofurantoin and fosfomycin. The majority were resistant to co-trimoxazole.

## Conclusions

Having emerged in 2017, OXA-244-Ec had been rather rare in Poland until 2023, when a dramatic increase occurred largely due to the regional outbreak in Lubuskie [7]. ST38 of both Clusters A and B [1] dominated among all 14 STs, being responsible for the regional and one single-hospital outbreaks. The entire study population demonstrated broad OXA-244-Ec dissemination in Poland. Moreover, its actual prevalence must have been underestimated, given problems with the Lubuskie outbreak control [7], and the lack of community isolates in the study in contrast to other countries. This might be attributed to the diagnostic difficulties with OXA-244-Ec [1], and relatively low numbers of microbiological examinations in the community in Poland, likely causing a largely unnoticed dissemination of the organism.

## Electronic supplementary material

Below is the link to the electronic supplementary material.

Supplementary Material 1

## Data Availability

Genomic sequences of all isolates have been deposited in the NCBI under the Bio-Projects numbers PRJNA1015038 and PRJNA1137236, and Bio-Samples SAMN37357215-55 and SAMN42934513-73, respectively.
