# Supplementary material for: Several epidemic and multiple sporadic genotypes of OXA-244-producing Escherichia coli in Poland; predominance of the ST38 clone
Source: Eur J Clin Microbiol Infect Dis. 2024 Oct 7;43(12):2465–72. doi: 10.1007/s10096-024-04954-0 (PMC11608361; doi:10.1007/s10096-024-04954-0)

Several epidemic and multiple sporadic genotypes of OXA-244-producing *Escherichia coli* in Poland; predominance of the ST38 clone

M. Biedrzycka,[1] R. Izdebski,[1] M. Gniadkowski,[1] and D. Żabicka[2*]

[1] *Department of Molecular Microbiology, National Medicines Institute, Chełmska 30/34, 00-725 Warsaw, Poland*

[2] *National Reference Centre for Susceptibility Testing, Department of Epidemiology and Clinical Microbiology, National Medicines Institute, Chełmska 30/34, 00-725 Warsaw, Poland*

* Corresponding author: Dorota Żabicka, d.zabicka@nil.gov.pl

Table S1. Resistomes of the OXA-244-producing *Escherichia coli* isolates representing distinguished profiles

Isolate	β-lactams	aminoglicosides	flouroquinolones	sulphonamides	trimethopirm	phenicols	tetracyclines	macrolides	profile	ST (n)
5550/23	*bla*$_{\text{CMY-2}}$, *bla*$_{\text{CTX-M-14}}$, *bla*$_{\text{CTX-M-231}}$, *bla*$_{\text{OXA-244}}$, *bla*$_{\text{TEM-1}}$	*aadA1, aph(3’)-Ib, aph(6)-Id, sat2*	*qnrS1*	*sul2*	*dfrA1*	-	-	*erm(B), mph(A)*	I	ST38 (35)
5553/23	*bla*$_{\text{CMY-2}}$, *bla*$_{\text{CTX-M-14}}$, *bla*$_{\text{OXA-244}}$, *bla*$_{\text{TEM-1}}$	*aadA1, aph(3’)-Ib, aph(6)-Id, sat2*	-	*sul2*	*dfrA1*	-	-	-	II	ST38 (12)
5558/23	*bla*$_{\text{CMY-2}}$, *bla*$_{\text{CTX-M-14}}$, *bla*$_{\text{CTX-M-231}}$, *bla*$_{\text{OXA-244}}$, *bla*$_{\text{TEM-1}}$	*aadA1, aph(3’)-Ib, aph(6)-Id, sat2*	*qnrS1*	-	*dfrA1*	-	-	*erm(B), mph(A)*	III	ST38 (1)
5578/23	*bla*$_{\text{CMY-2}}$, *bla*$_{\text{CTX-M-14}}$, *bla*$_{\text{CTX-M-231}}$, *bla*$_{\text{OXA-244}}$, *bla*$_{\text{TEM-1}}$	*aadA1, aph(3’)-Ib, aph(6)-Id, sat2*	-	*sul2*	*dfrA1*	-	-	*erm(B), mph(A)*	IV	ST38 (1)
5579/23	*bla*$_{\text{CMY-2}}$, *bla*$_{\text{CTX-M-14}}$, *bla*$_{\text{CTX-M-231}}$, *bla*$_{\text{OXA-244}}$, *bla*$_{\text{TEM-1}}$	*aadA1, aadA5, aac(3)-IId, aph(3’)-Ib, aph(6)-Id, sat2*	*qnrS1*	*sul1, sul2*	*dfrA1, dfrA17*	-	-	*erm(B), mph(A)*	V	ST38 (1)
2213/18	*bla*$_{\text{CTX-M-14}}$, *bla*$_{\text{OXA-244}}$, *bla*$_{\text{TEM-1}}$	*aadA1, aph(3’)-Ia, aph(3’’)-Ib, aph(6)-Id, sat2*	-	*sul2*	*dfrA1*	-	*tet(D)*	-	VI	ST38 (1)
8934/17	*bla*$_{\text{CTX-M-27}}$, *bla*$_{\text{OXA-244}}$	*aadA5, aph(3’’)-Ib, aph(6)-Id*	-	*sul1, sul2*	*dfrA17*	-	*tet(A)*	*mph(A)*	VII	ST38 (11)
8517/18	*bla*$_{\text{CTX-M-14}}$, *bla*$_{\text{OXA-244}}$, *bla*$_{\text{TEM-1}}$	*aadA1, aph(3’)-Ia, aph(3’’)-Ib, aph(6)-Id*		*sul2*	*dfrA1*	-	-	-	VIII	ST38 (2)
6093/23	*bla*$_{\text{CTX-M-27}}$, *bla*$_{\text{OXA-244}}$	*aph(3’’)-Ib, aph(6)-Id*	-	*sul2*	-	-	*tet(A)*	-	IX	ST38 (1)
6094/23	*bla*$_{\text{CTX-M-14}}$, *bla*$_{\text{OXA-244}}$, *bla*$_{\text{TEM-1}}$	*aadA1, aph(3’’)-Ib, aph(6)-Id, sat2*	-	*sul2*	*dfrA1*	-	-	-	X	ST38 (2)
6227/23	*bla*$_{\text{CMY-2}}$, *bla*$_{\text{CTX-M-14}}$, *bla*$_{\text{OXA-244}}$, *bla*$_{\text{TEM-1}}$	*aadA1, aph(3’’)-Ib, aph(6)-Id, sat2*	-	*sul2*	*dfrA1*	-	-	*mph(A)*	XI	ST38 (1)
7712/23	*bla*$_{\text{CMY-2}}$, *bla*$_{\text{OXA-244}}$, *bla*$_{\text{TEM-1}}$	*aadA5, aac(3)-IId, aph(3’’)-Ib, aph(6)-Id*	-	*sul1, sul2*	*dfrA17*	-	-	*mph(A)*	XII	ST38 (1)
9336/23	*bla*$_{\text{CTX-M-14}}$, *bla*$_{\text{OXA-244}}$, *bla*$_{\text{TEM-1}}$	*aadA1, aph(3’’)-Ib, aph(6)-Id, sat2*	-	*sul2*	*dfrA1*	-	*tet(D)*	-	XIII	ST38 (1)
6283/23	*bla*$_{\text{OXA-244}}$, *bla*$_{\text{TEM-1}}$	*aadA5, aph(3’’)-Ib, aph(6)-Id*	-	*sul1, sul2*	*dfrA17*	-	*tet(A)*	*mph(A)*	XIV	ST58 (9)
1527/23	*bla*$_{\text{CTX-M-15}}$, *bla*$_{\text{OXA-244}}$	-	*qnrS1*	-	-	-	-	-	XV	ST10 (1)
6988/23	*bla*$_{\text{CTX-M-14}}$, *bla*$_{\text{OXA-244}}$	-	-	-	-	-	-	-	XVI	ST10 (1)
8494/23	*bla*$_{\text{OXA-244}}$	-	-	-	-	-	-	-	XVII	ST10 (2), ST69 (1), ST442 (1)
9465/23	*bla*$_{\text{CTX-M-15}}$, *bla*$_{\text{OXA-244}}$	*aadA5, aph(3’’)-Ib, aph(6)-Id*	-	*sul1, sul2*	*dfrA17*	-	*tet(A)*	*mph(A)*	XVIII	ST69 (1)
9537/23	*bla*$_{\text{CTX-M-15}}$, *bla*$_{\text{OXA-244}}$	*aadA5*	*qnrS1*	*sul1*	*dfrA17*	-	-	*erm(B), mph(A)*	XIX	ST69 (1)
1067/22	*bla*$_{\text{CMY-145}}$, *bla*$_{\text{OXA-244}}$	*aadA2*	-	*sul1*	*dfrA12*	-	-	*erm(B), mph(A)*	XX	ST167 (1)
8254/22	*bla*$_{\text{CTX-M-15}}$, *bla*$_{\text{OXA-244}}$	*aadA5, aph(3’’)-Ib, aph(6)-Id*	*qnrS1*	*sul1, sul2*	*dfrA17*	-	*tet(B)*	*erm(B), mph(A)*	XXI	ST167 (2), ST361 (1)
7476/21	*bla*$_{\text{OXA-244}}$, *bla*$_{\text{NDM-5}}$	*aadA2*	-	*sul1*	*dfrA12*	-	*tet(A)*	-	XXII	ST361 (1)
8342/23	*bla*$_{\text{CTX-M-15}}$, *bla*$_{\text{OXA-244}}$	*aadA5*	*qnrS1*	*sul1*	*dfrA17*	-	*tet(B)*	-	XXIII	ST361 (1)
5887/21	*bla*$_{\text{CTX-M-15}}$, *bla*$_{\text{OXA-244}}$	*aadA5*	-	*sul1*	*dfrA17*	-	-	*erm(B), mph(A)*	XXIV	ST131 (2)
10056/21	*bla*$_{\text{OXA-244}}$, *bla*$_{\text{TEM-1}}$	-	-	-	-	*catA1*	-	-	XXV	ST224 (1)
7920/23	*bla*$_{\text{CMY-2}}$, *bla*$_{\text{CTX-M-15}}$, *bla*$_{\text{OXA-1}}$, *bla*$_{\text{OXA-244}}$, *bla*$_{\text{TEM-1}}$	*aadA5, aac(3)-IId, aph(3’’)-Ib, aph(6)-Id, aac(6’)-Ib-cr*	*aac(6’)-Ib-cr*	*sul1, sul2*	*dfrA17*	*catA2*	*tet(B)*	*mph(A)*	XXVI	ST410 (1)
8174/23	*bla*$_{\text{OXA-244}}$, *bla*$_{\text{TEM-1}}$	*aadA5, aac(3)-IId*	-	-	*dfrA17*	-	-	*mph(A)*	XXVII	ST2178 (1)
6226/23	*bla*$_{\text{DHA-1}}$, *bla*$_{\text{OXA-244}}$	-	*qnrB4*	*sul1*	*dfrA17*	-	-	*mph(A)*	XXVIII	ST2346 (1)
1047/21	*bla*$_{\text{CTX-M-27}}$, *bla*$_{\text{OXA-244}}$	-	-	-	*dfrA14*	-	*tet(B)*	*mph(A)*	XXIX	ST3541 (1)
8386/23	*bla*$_{\text{CTX-M-15}}$, *bla*$_{\text{OXA-244}}$	-	-	-	*dfrA14*	-	*tet(B)*	*mph(A)*	XXX	ST13730 (1)

Table S2. Resistomes of the isolates subjected to the long-read WGS analysis

Isolate	Chromosome	Plasmids
5550/23	*bla*$_{CMY-2}$, *bla*$_{CTX-M-14,}$ *bla*$_{OXA-244,}$ *bla*$_{TEM-1}$, *aadA1, aph(3')-Ib, aph(6)-Id, sat2, sul2, dfrA1*	**IncFII-like (85518 bp):** *bla*$_{CTX-M-231,}$ *bla*$_{TEM-1}$, *qnrS1, erm(B), mph(A)*
1506/20	*bla*$_{OXA-244}$	**IncFII-like (102120 bp):** *bla*$_{CTX-M-27,}$ *aadA5, aph(3'')-Ib, aph(6)-Id, sul1, sul2, dfrA17, tet(A), mph(A)*
6864/23	*bla*$_{OXA-244}$	**IncFII-like (128880 bp):** *bla*$_{TEM-1}$, *aadA5, aph(3'')-Ib, aph(6)-Id, sul1, sul2, dfrA17, tet(A), mph(A)*

Table S3. Antimicrobial susceptibility of the OXA-244-producing *Escherichia coli* isolates representing distinguished profiles

Isolate ID (AMR profile)	AMP	AMC	TZP	TEM	CTX	CAZ	FEP	FDC	ATM	IMP	MEM	ERT	CZA	MVB	IPR	CIP	LEV	AMK	GEN	TOB	TIG	ERV	CST	NIT	SXT	FOS
5550/23 (I)	**>16**	**>32**	**>64**	**>64**	**>32**	**>16**	**>16**	1	**32**	≤1	2	**>2**	0.5	1	0.5	0.25	0.5	4	1	1	≤0.25	0.12	≤0.25	≤32	**>8**	1
5553/23 (II)	**>16**	**>32**	**>64**	**>64**	**>32**	**16**	**16**	0.25	**32**	≤1	0.5	**>2**	≤0.25	0.5	0.25	0.25	≤0.25	≤2	≤0.5	≤0.5	≤0.25	0.12	≤0.25	≤32	**>8**	1
5558/23 (III)	**>16**	**>32**	**>64**	**>64**	**>32**	**>16**	**>16**	0.5	**32**	≤1	1	**>2**	≤0.25	0.5	0.25	0.25	0.5	≤2	≤0.5	≤0.5	≤0.25	0.12	≤0.25	≤32	**>8**	1
5578/23 (IV)	**>16**	**>32**	**>64**	**>64**	**>32**	**>16**	**16**	0.5	**32**	≤1	0.5	**>2**	≤0.25	0.5	0.25	0.25	0.5	4	≤0.5	≤0.5	≤0.25	0.12	≤0.25	≤32	**>8**	1
5579/23 (V)	**>16**	**>32**	**>64**	**>64**	**>32**	**16**	**16**	0.25	**32**	≤1	0.5	**>2**	≤0.25	0.5	0.25	0.25	0.5	≤2	≤0.5	≤0.5	≤0.25	0.12	≤0.25	≤32	**>8**	1
2213/18 (VI)	**>16**	**>32**	**>64**	**>64**	**>32**	*4*	**16**	0.25	**16**	≤1	0.5	**2**	0.5	0.5	0.25	≤0.06	≤0.25	≤2	≤0.5	≤0.5	≤0.25	0.06	≤0.25	≤32	**>8**	1
8934/17 (VII)	**>16**	**>32**	**>64**	**>64**	**>32**	**8**	**16**	0.5	**8**	4	**16**	**>2**	≤0.25	8	**4**	≤0.06	≤0.25	≤2	≤0.5	≤0.5	≤0.25	0.25	≤0.25	≤32	**>8**	2
8517/18 (VIII)	**>16**	**>32**	**>64**	**>64**	**>32**	*4*	**>16**	0.5	**16**	≤1	1	**>2**	≤0.25	1	0.5	≤0.06	≤0.25	≤2	1	1	≤0.25	0.25	≤0.25	≤32	**>8**	1
6093/23 (IX)	**>16**	**>32**	**>64**	**>64**	**>32**	**8**	**8**	0.5	**16**	≤1	2	**>2**	≤0.25	0.5	0.25	≤0.06	≤0.25	≤2	≤0.5	≤0.5	≤0.25	0.25	≤0.25	≤32	≤0.5	0.5
6094/23 (X)	**>16**	**>32**	**>64**	**>64**	**>32**	**16**	**16**	0.5	**16**	≤1	1	**2**	≤0.25	0.5	0.25	≤0.06	≤0.25	≤2	≤0.5	≤0.5	≤0.25	0.12	≤0.25	≤32	**>8**	1
6227/23 (XI)	**>16**	**>32**	**>64**	**>64**	**>32**	**>16**	**>16**	2	**32**	≤1	1	**>2**	≤0.25	0.5	0.5	≤0.06	≤0.25	≤2	≤0.5	≤0.5	≤0.25	0.12	≤0.25	≤32	**>8**	1
7712/23 (XII)	**>16**	**>32**	**>64**	**>64**	**8**	**8**	≤0.5	0.125	*4*	≤1	0.5	**>2**	≤0.25	0.5	0.25	≤0.06	≤0.25	4	**>8**	**8**	≤0.25	0.12	≤0.25	≤32	**>8**	1
9336/23 (XIII)	**>16**	**>32**	**>64**	**>64**	**>32**	*4*	**>16**	0.5	**16**	≤1	0.5	**2**	≤0.25	0.25	0.25	≤0.06	≤0.25	≤2	≤0.5	≤0.5	≤0.25	0.12	≤0.25	32	**>8**	1
6283/23 (XIV)	**>16**	**>32**	**>64**	**>64**	≤0.5	≤0.5	≤0.5	0.5	≤1	≤1	0.25	0.5	≤0.25	≤0.06	0.25	≤0.06	≤0.25	4	≤0.5	1	≤0.25	0.25	≤0.25	≤32	**>8**	0.5
1527/23 (XV)	**>16**	**>32**	**>64**	**>64**	**32**	*2*	1	1	*4*	≤1	≤0.12	0.5	≤0.25	≤0.06	0.25	0.25	≤0.25	≤2	≤0.5	≤0.5	≤0.25	0.12	≤0.25	≤32	≤0.5	1
6988/23 (XVI)	**>16**	**>32**	**64**	**>64**	**32**	≤0.5	*2*	0.5	≤1	≤1	≤0.12	0.5	≤0.25	≤0.06	0.25	≤0.06	≤0.25	≤2	≤0.5	≤0.5	≤0.25	0.25	≤0.25	≤32	≤0.5	1
8494/23 (XVII)	**>16**	**>32**	**>64**	**>64**	≤0.5	≤0.5	≤0.5	0.5	≤1	≤1	0.5	**1**	≤0.25	0.12	0.25	≤0.06	≤0.25	≤2	≤0.5	≤0.5	≤0.25	0.25	≤0.25	≤32	≤0.5	2
9465/23 (XVIII)	**>16**	**>32**	**>64**	**>64**	**16**	*2*	1	0.5	**8**	≤1	≤0.12	0.25	≤0.25	0.12	0.12	0.25	0.5	≤2	≤0.5	≤0.5	≤0.25	0.25	≤0.25	≤32	**>8**	1
9537/23 (XIX)	**>16**	**>32**	**>64**	**>64**	**32**	*2*	*2*	0.5	*4*	≤1	≤0.12	0.5	≤0.25	0.12	0.25	0.25	0.5	≤2	≤0.5	≤0.5	≤0.25	0.12	≤0.25	≤32	**>8**	1
1067/22 (XX)	**>16**	**>32**	**>64**	**>64**	**>32**	**>16**	**8**	**4**	**>32**	≤1	0.5	**>2**	2	0.5	0.25	**>2**	**>8**	4	≤0.5	≤0.5	≤0.25	0.12	≤0.25	≤32	**>8**	0.5
8254/22 (XXI)	**>16**	**>32**	**>64**	**>64**	**>32**	**>16**	**>16**	**4**	**>32**	≤1	0.25	**2**	0.5	0.12	0.25	**>2**	**>8**	≤2	≤0.5	≤0.5	≤0.25	0.25	≤0.25	≤32	**>8**	0.5
7476/21 (XXII)	**>16**	**>32**	**>64**	**>64**	**>32**	**>16**	**>16**	2	*2*	**>8**	**>16**	**>2**	**>16**	**>16**	**>8**	**>2**	**>8**	≤2	≤0.5	≤0.5	≤0.25	0.5	≤0.25	≤32	**>8**	1
8342/23 (XXIII)	**>16**	**>32**	**>64**	**>64**	**>32**	**>16**	**>16**	2	**>32**	≤1	0.25	**>2**	2	0.25	0.25	**>2**	**>8**	≤2	≤0.5	≤0.5	≤0.25	0.12	≤0.25	64	**>8**	1
5887/21 (XXIV)	**>16**	**>32**	**64**	**32**	**>32**	**8**	**8**	0.5	**32**	≤1	0.25	0.5	≤0.25	0.12	0.25	**1**	*1*	≤2	≤0.5	1	≤0.25	0.25	≤0.25	≤32	**>8**	1
10056/21 (XXV)	**>16**	**>32**	**>64**	**>64**	≤0.5	≤0.5	≤0.5	0.125	≤1	≤1	0.25	**1**	≤0.25	0.25	0.5	**>2**	**>8**	≤2	1	2	≤0.25	0.25	≤0.25	≤32	≤0.5	2
7920/23 (XXVI)	**>16**	**>32**	**>64**	**>64**	**>32**	**>16**	**>16**	2	**>32**	≤1	0.5	**>2**	0.5	0.25	0.25	**>2**	**>8**	4	2	**>8**	≤0.25	0.12	≤0.25	64	**>8**	1
8174/23 (XXVII)	**>16**	**>32**	**>64**	**64**	≤0.5	≤0.5	≤0.5	0.125	≤1	≤1	0.25	**1**	≤0.25	0.25	0.25	0.25	0.5	4	**>8**	**8**	≤0.25	0.12	≤0.25	≤32	≤0.5	0.5
6226/23 (XXVIII)	**>16**	**>32**	**>64**	**>64**	**4**	**8**	≤0.5	0.5	≤1	≤1	0.5	**1**	≤0.25	1	0.25	0.25	≤0.25	≤2	≤0.5	1	≤0.25	0.25	≤0.25	≤32	1	1
1047/21 (XXIX)	**>16**	**>32**	**>64**	**>64**	**>32**	**>16**	**16**	0.5	**32**	≤1	0.5	**>2**	≤0.25	0.5	0.25	≤0.06	≤0.25	≤2	1	≤0.5	≤0.25	0.12	≤0.25	≤32	1	0.5
8386/23 (XXX)	**>16**	**>32**	**>64**	**>64**	**>32**	**16**	**>16**	1	**32**	≤1	0.25	**1**	≤0.25	0.12	0.5	**1**	*1*	4	≤0.5	≤0.5	≤0.25	0.12	≤0.25	≤32	1	0.5

AMC: amoxicillin-clavulanic acid; AMK: amikacin; AMP: ampicillin; ATM: aztreonam; CAZ: ceftazidime; CIP: ciprofloxacin; CST: colistin; CTX: cefotaxime; CZA: ceftazidime-avibactam; ERT: ertapenem; ERV: eravacycline; FDC: cefiderocol; FEP: cefepime; FOS: fosfomycin; GEN: gentamicin; IMP: imipenem; IPR: imipenem-relebactam; LEV: levofloxacin; MEM: meropenem; MVB: meropenem-vaborbactam; NIT: nitrofurantoin; SXT: trimethoprimsulfamethoxazole; TEM: temocillin; TIG: tigecycline; TOB: tobramycin; TZP: piperacillin-tazobactam. Resistant isolates are marked with bold, isolates defined as susceptible, increased exposure, are marked with italic.

Figure S1. Annual distribution of all OXA-48-type-Ec identified in Poland since 2013 (n=244), with the contribution of OXA-244-Ec isolates indicated

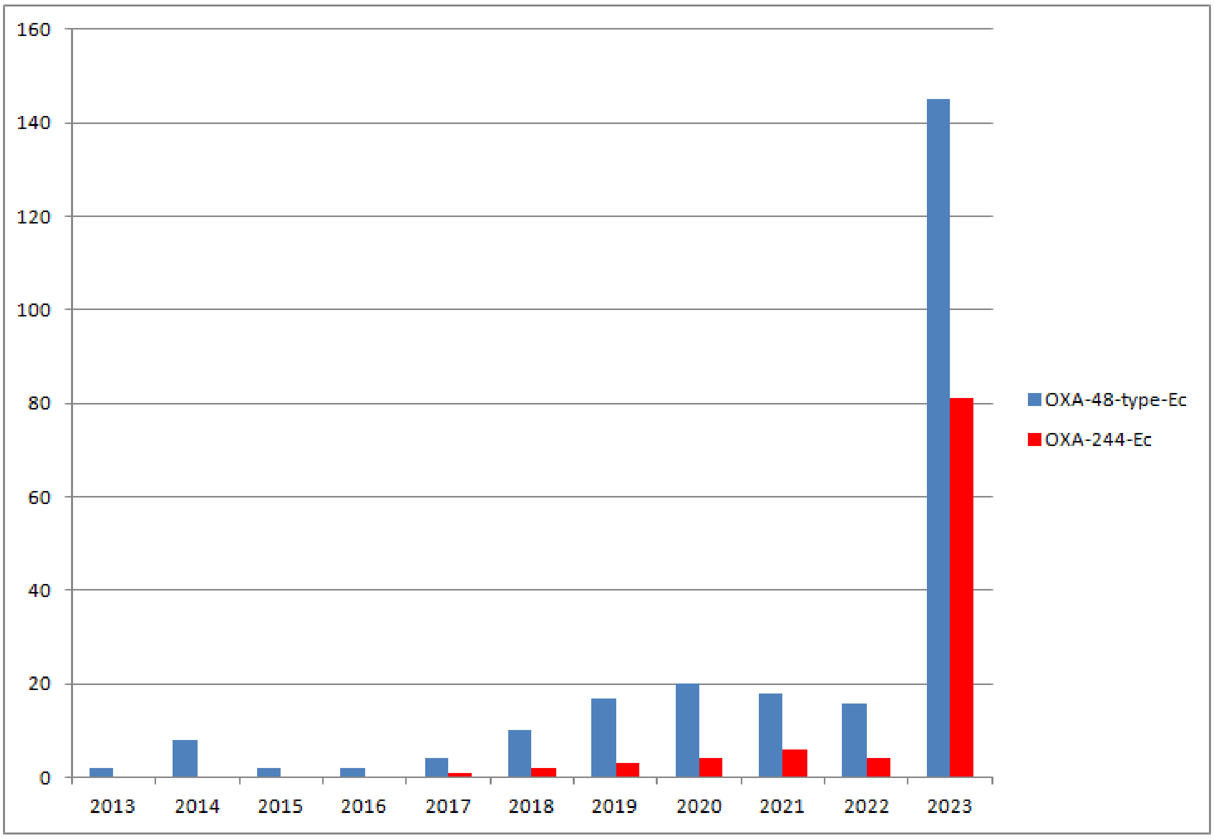

Supplement: Supplementary file 1 — Supplementary Material 1 [file 10096_2024_4954_MOESM1_ESM.pdf]
